# Nose-to-Brain Delivery of Therapeutic Peptides as Nasal Aerosols

**DOI:** 10.3390/pharmaceutics14091870

**Published:** 2022-09-05

**Authors:** Wafaa Alabsi, Basanth Babu Eedara, David Encinas-Basurto, Robin Polt, Heidi M. Mansour

**Affiliations:** 1 Skaggs Pharmaceutical Sciences Center, College of Pharmacy, The University of Arizona, 1703 E. Mabel St, Tucson, AZ 85721, USA; 2 Department of Chemistry and Biochemistry, The University of Arizona, Tucson, AZ 85721, USA; 3 Center for Translational Science, Florida International University, Port St. Lucie, FL 34987, USA; 4 The BIO5 Institute, The University of Arizona, Tucson, AZ 85721, USA; 5 Department of Medicine, Division of Translational and Regenerative Medicine, The University of Arizona College of Medicine, Tucson, AZ 85721, USA

**Keywords:** peptides, intranasal delivery, blood-brain barrier (BBB), nebulization, central nervous system (CNS), dry powders, nasal devices

## Abstract

Central nervous system (CNS) disorders, such as psychiatric disorders, neurodegeneration, chronic pain, stroke, brain tumor, spinal cord injury, and many other CNS diseases, would hugely benefit from specific and potent peptide pharmaceuticals and their low inherent toxicity. The delivery of peptides to the brain is challenging due to their low metabolic stability, which decreases their duration of action, poor penetration of the blood-brain barrier (BBB), and their incompatibility with oral administration, typically resulting in the need for parenteral administration. These challenges limit peptides’ clinical application and explain the interest in alternative routes of peptide administration, particularly nose-to-brain (N-to-B) delivery, which allows protein and peptide drugs to reach the brain noninvasively. N-to-B delivery can be a convenient method for rapidly targeting the CNS, bypassing the BBB, and minimizing systemic exposure; the olfactory and trigeminal nerves provide a unique pathway to the brain and the external environment. This review highlights the intranasal delivery of drugs, focusing on peptide delivery, illustrating various clinical applications, nasal delivery devices, and the scope and limitations of this approach.

## 1. Introduction

Endogenous neuropeptides perform complex and specific modulatory functions [1]. They are of increasing interest to the pharmaceutical industry because of their potential advantages as therapeutic molecules [1,2]. The peptide therapeutics market globally was valued at US$25 billion in 2018, is expected to reach US$49.5 billion by 2027 [3]. The key advantages of therapeutic peptides include their high potency, selectivity, minimum side effects, specificity for their target receptors, minimal drug-drug interactions, and reduced immunogenicity [1]. Furthermore, they are biodegraded efficiently into nontoxic metabolites by endogenous enzymes; thus, peptide hormones do not accumulate in tissues [1,2]. As a result, these favorable properties enable peptide drugs to secure quicker regulatory approval than small molecule drugs [2]. However, despite the excellent pharmacological properties of peptides, their poor physical and metabolic stability still present significant challenges for efficient delivery; thus, their main limitation as therapeutics candidates is the short half-life in vivo. Many peptides are cleared from the bloodstream shortly after administration resulting in limited exposure to the brain capillaries, reducing their ability to penetrate the BBB. The short half-life generally results from rapid renal clearance, enzymatic digestion by proteases, or both [1].

Orally administered peptides are poorly absorbed and rapidly destroyed by the stomach’s low pH and digestion of gastrointestinal enzymes. Thus, there is typically a need for parenteral administration of peptides [1,2,4]. However, the parenteral route of administration has several drawbacks, since it is invasive, painful, and poorly accepted by patients, especially when prolonged or repeated use is required [5]. Another obstacle to peptide therapeutics is their limited ability to cross biological membranes and cellular barriers [2]; considering the brain and the CNS specifically, BBB penetration rates and the P-glycoprotein (P-gp) efflux pump are critical factors limiting their application in the clinic [1].

The BBB comprises endothelial cells cemented to each other through tight endothelial capillary cell junctions; the capillaries are surrounded by astrocyte feet and efflux pumps on the endothelial cells, and degradative enzymes close to the abluminal surface [6]. The tight junctions of the BBB resulted in a higher transendothelial electric resistance (1500–2000 Ω·cm^2^) compared to that of other tissues (3–33 Ω·cm^2^). The second defense mechanism includes the multidrug efflux protein transporters. It is evaluated that half of all drug candidates are substrates for the P-gp efflux pump, presenting a reduced potential for systemic penetration to the CNS [7]. 

Furthermore, the short plasma half-life of most peptides means that few peptides show any actual bioavailability in the brain unless transported via specific carriers. Certainly, the peptides’ delivery to the brain today would require invasive techniques such as intracerebral injections, intracerebroventricular infusion, and convection-enhanced delivery. Or otherwise, using the intracranial implants, or temporary disruption of the BBB using osmotic agents or ultrasound [2,8,9,10,11]. Nevertheless, these methodologies are associated with a high risk of infections, inflammation, high intracranial pressure, and damage to the brain tissue, and most of them are not suitable for chronic treatments [8,11].

The several possible routes by which peptides could penetrate the BBB are diffusion, receptor-mediated endocytosis, and carrier-mediated uptake. Enhanced lipophilicity is required for diffusion, while for receptor-mediated and carrier-mediated uptake, a transport-specific ligand must be incorporated within the peptide medicine. Weak hydrogen bonding potential, lipophilicity, and small molecular size, with the absence of free rotatable bonds and a polar surface area of <60−70 Å (being largely apolar), are favorable for permeation across the BBB via diffusion. Thus, passive diffusion of peptides is very limited unless the peptides possess an amphipathic structure or use synthetic means to modify the lipophilicity [2,6], such as the glycosylation strategy of peptides [12]. Glycopeptides have relatively lipophilic backbones and hydrophilic sugar moieties; this allows a glycopeptide to reversibly interact with the membrane and aqueous compartment in a “hopping” motion, promoting transport of the glycopeptide across the membranes, including BBB [12,13,14].

The BBB presence is the primary limiting factor for drug delivery to the brain through the systemic circulation. Several methods are being developed to bypass the BBB; among these approaches is N-to-B delivery which is becoming of great interest as an alternative to usual administration routes [4,6,7,8,11,13,14].

N-to-B delivery is a non-invasive method that enables drug delivery to the CNS, bypassing the BBB through the only place that directly connects the CNS with the environment [9,15,16]. By this route, the drugs can rapidly access the CNS following a ‘‘shortcut” from the nose to the brain, directly utilizing trigeminal or olfactory nerves located in the upper part of the nasal cavity [10,16]. A vital advantage of the N-to-B route is reducing plasma exposure, decreasing peripheral side effects, and avoiding hepatic first-pass metabolism and the destructive effects of gastric acid. [6,17,18]. As a result, lower drug administration requirements are feasible [17], so no modification of the therapeutic agent nor coupling the drug to any carrier is required [11]. The nasal drug delivery route includes local and systemic drug delivery [18] and can deliver various therapeutic agents, including macromolecules and small molecules to the CNS [11]. Thus, intranasal (IN) drug delivery offers a non-invasive direct, effective in smaller doses, and alternative route to the CNS; moreover, it is convenient for long-term chronic therapy in patients [11,18].

This review focuses on advances in therapeutic peptide brain delivery as liquid formulations and dry powder inhalers (DPIs), besides highlighting the role of IN-delivered high molecular weight molecules (including peptides) in treating various systemic diseases. Additionally, this review briefly highlights the anatomy of the nasal route, pathways, and mechanisms underlying IN peptide delivery to the CNS. Furthermore, this review covers a detailed description of the nasal delivery devices; nasal liquid formulated peptides for CNS therapy and disease application; nasal DPI peptides for CNS therapy disease application; and clinical trials of nasal liquid formulations and nasal DPIs for the CNS.

## 2. Nasal Anatomy

A summary of nasal anatomy helps understand the mechanism of IN therapy [17]. The nasal cavity’s primary functions are breathing, olfaction, protective activity by warming and humidifying inspired air before reaching the lowest airways, removing inhaled particles, and modifying airflow [4,7]. Anatomically, the human nasal cavity is in the space between the base of the skull and the roof of the mouth, below the cribriform plate of the ethmoid bone, which supports it and separates the nasal and cranial cavities [16,19]. Humans’ nasal cavity has a total surface area of approximately 150 cm^2^ and a total volume of 15–20 mL [4,7]. It is divided symmetrically by the middle septum into two halves, opening at the face through the nostrils and extending posteriorly to the nasopharynx. These symmetrical halves contain four areas distinguished according to their anatomic and histological characteristics: nasal vestibule, atrium, respiratory region, and olfactory region [7]. In other words, the nasal vestibule, the respiratory region, and the olfactory region are the three main regions of the nasal cavity [17,19], with the olfactory situated high up in the nares and the vestibule closer to the nostrils [6]. The nasal epithelium is well vascularized, and, within the olfactory area, olfactory neurons are exposed, enabling compounds’ transportation into the brain via the olfactory neurons [6].

The olfactory tissue is often yellow in humans, while the surrounding tissues are pink. The olfactory epithelial layer contains three cell types: the olfactory neural cells, the subtentacular cells, and the basal cells [16,19]. Basal cells are progenitor cells anchored to other cells providing mechanical support, and the olfactory epithelium is a gateway for substances entering the CNS and peripheral circulation [19]. The olfactory region comprises just 3–5% of the total nasal cavity surface area yet is the most critical anatomical region regarding IN drug administration [17].

## 3. Mechanism of Nasal Drug Delivery to Brain

The exact mechanisms of IN drug delivery to the CNS are not completely understood [6,8]; however, much evidence shows that pathways involving nerves connecting the nasal passages to the brain and spinal cord are essential. In addition, pathways involving the cerebrospinal fluid (CSF), vasculature, and lymphatic system have been implicated in transporting molecules from the nasal cavity to the CNS [8]. For a peptide to go to the CSF or brain tissues from the olfactory area of the nasal cavity, it must cross the olfactory nasal epithelium [2]. Three different possible pathways across the olfactory epithelium exist: (a) a paracellular pathway via tight junctions between the sustentacular cells and olfactory neurons; (b) a transcellular pathway, mainly across the sustentacular cells, most likely by receptor-mediated endocytosis, fluid-phase endocytosis, or passive diffusion (not for peptides); and (c) the olfactory nerve pathway where the drug is taken up into the neurons by endocytotic or pinocytotic mechanisms and transported by intracellular axonal transport to the olfactory bulb [2,16].

The paracellular pathway is considered the dominant transport mechanism based on animal studies. It allows faster drug transport than the others (usually <30 min) that lasts from a few hours up to days. An explanation for this could be the slow regeneration of the olfactory neurons, leading to the coexistence of mature and newly formed neurons; thus, the tight junctions will disappear in some parts of the olfactory epithelium. This leakiness, combined with the CSF’s bulk flow into the brain, enables transport of the intranasally administered compounds to the CNS [16]. However, a combination of multiple pathways is likely responsible, although one pathway may predominate, depending on the properties of the compound, the characteristics of the formulation, and the delivery device used [8]. Depending on the pathway, the drug may reach the olfactory bulb via intraneuronal uptake; then, it may go into the brain regions that contact the olfactory tract, such as the piriform cortex, hypothalamus, and amygdala. Finally, it diffuses directly from the CSF and spreads into the whole CNS [16]. Drugs that usually cannot enter the CNS may become therapeutically beneficial by IN administration, as they bypass the BBB at significantly lower doses, with a subsequent decrease in adverse effects [9]. Systemic drug delivery complications are theoretically minimized because inhaled drugs are not administered systemically [17]. Thus, drugs avoid the hepatic first-pass effect and/or degradation in the bloodstream, which is a crucial issue in the case of peptide drugs [20,21].

## 4. Limitations and Challenges of Nasal Drug Delivery to Brain

Despite the potential of the N-to-B drug delivery route to the CNS, there are significant challenges associated with this method of administration. N-to-B transport is significantly affected by the administered biomolecules’ surface and structural properties such as size, lipophilicity, and degree of ionization. Another critical factor is the metabolic enzymes in the mammalian olfactory mucosa. Anatomically, the olfactory epithelium localization in the nasal cavity roof leads to difficult drug access to the targeted region. This route’s limitation is related to the small dose that can be administered, which implies the need to design and formulate a drug with a high loading capacity [16], making the route effective only for very potent drugs [6]. Despite the above-listed limitations, several advantages of this pain-free, non-invasive, and direct administration are obvious; therefore, delivering the peptides via this N-to-B strategy is a potential alternative to the other CNS delivery strategies [9,16]. Furthermore, the decreasing number of low molecular weight drugs being approved for clinical use has increased the efforts to develop peptides as potential successful therapeutics [2].

## 5. Intranasal Formulations

The targeted delivery of drugs from N-to-B is affected by many factors, including the dosage form choice. The selection of a suitable dosage form mainly depends on the physicochemical nature of the drug molecules, therapeutic aim, patient compliance, and marketing issues. IN drug formulations are categorized into liquid, semi-solid, and powder formulations.

### 5.1. Liquid Formulations

Nasal liquid formulations are the most used dosage forms. They are mostly aqueous solutions, suspensions, or emulsions [22]. They act as humectants and prevent dryness of the nasal mucosal membrane, associated with several allergic and chronic disease conditions [23]. For nasal formulations, additional controls should be applied to excipients to ensure the effectiveness and safety of the final product. For example, the excipients for suspension formulations can affect the suspension and the particle characteristics, thus, the drug product’s quality, stability, and performance [24]. 

Generally, liquid formulations are administered at a dose volume of 25–150 µL, with an upper limit of 200 µL [25]. The major disadvantages of the aqueous liquid formulations are microbial contamination, chemical instability of the dissolved drug in aqueous conditions, presence of preservatives that may cause mucosal irritation, and short residence time in the nasal cavity due to mucociliary action [21]. In addition, maintaining the stability of protein and peptide drugs in the aqueous media is a more significant challenge in IN liquid formulations. It requires stabilizing agents and proper storage conditions to maintain the shelf life of the product. Liquid formulations are delivered using various devices (discussed in detail in the following section) such as catheters, droppers, squeeze bottles, metered-dose spray pumps, pressurized metered-dose inhalers, pressurized olfactory devices, nebulizers, and atomizers.

### 5.2. Semi-Solid Formulations

Semi-solid formulations include nasal gels, thermo-responsive or pH-sensitive or ion-sensitive polymeric gels, and ointments. They are vicsous and can enhance the drug residence time in the nasal cavity, which improves the IN delivery of drugs to the brain. The thermo-responsive gel system consists of polymers that undergo sol-gel phase transformation in a temperature range of 25–37 °C. Some of the most common thermo-responsive polymers used to prepare IN gel formulations include poloxamers (poloxamer 188 and poloxamer 407) and chitosan [26,27]. Upon IN administration, the polymer moieties get arranged into micellar packing and form a viscous gel at the body temperature. The pH-sensitive gels consist of polymers such as Carbopol 934, and Carbopol 940 maintained at a lower pH ranging from 4 to 5.5. Upon IN administration, they undergo sol-gel phase transformation at the nasal pH of ~6.2 and form a three-dimensional gel structure [28,29,30]. Ion-sensitive polymeric systems demonstrate sol-gel transitions due to ionic stimulus in the physiological environment. Gellan gum and pectin are the most commonly used natural polysaccharides used as ion-sensitive gelling agents for nasal formulations. They both undergo gelation via cationic complexation with their functional groups and result in 3-dimensional network formation [30]. However, the viscous semi-solid formulations may cause nasal obstruction during IN administration and reduce the nasal cavity’s formulation spreadability [31].

### 5.3. Dry Powder Formulations

**Figure 1:**
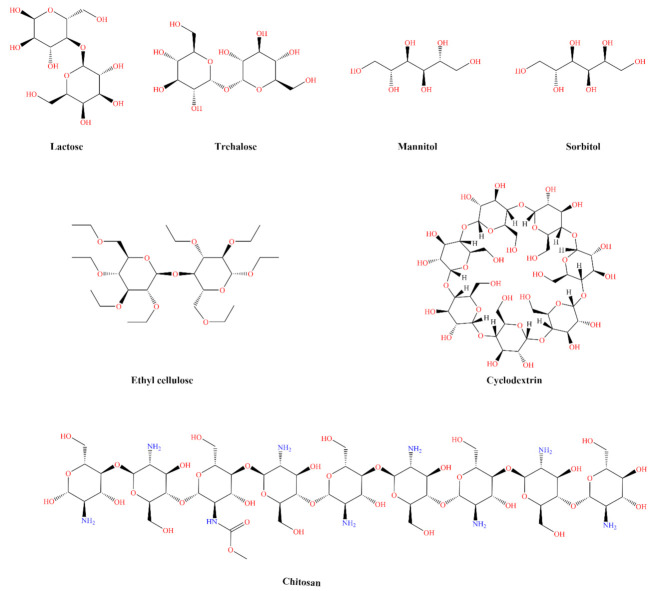
Chemical structures (drawn using CambridgeSoft^™^, Cambridge, MA, USA) of some examples of excipients used in powder formulations of protein and peptide.

Most IN formulations are in liquid form. However, dry powder formulations have attracted greater attention due to the dry powder particle’s greater drug stability and prolonged residence time in the nasal cavity compared to liquids, and also due to the potential to achieve the desired therapeutic drug levels in the brain by controlling the release rate of encapsulated drug [32]. Further, for poorly water-soluble drugs and biopharmaceuticals, such as proteins and peptides, powder formulations facilitate the formulation of larger drug doses by co-formulating using excipients that act as bulking agents (water-soluble fillers: lactose, trehalose, mannitol, and sorbitol; water-insoluble fillers: talc, calcium carbonate, barium sulfate, or ethylcellulose), mucoadhesive agents (starch, chitosan, gelatin, starch, cellulose derivatives), solubilizers, permeation and absorption enhancers (cyclodextrins, fatty acids, phospholipids, surfactants, bile salts, chelating agents) for better therapeutic efficacy [13,14,32,33,34,35,36]. Figure 1 shows chemical structures of some excipients used in powder formulations of protein and peptide. Generally, IN dry powder formulations are manufactured using solvent evaporation, emulsification solvent evaporation, freeze-drying, spray drying, and spray freeze-drying techniques. 

## 6. Nasal Delivery Devices

Different administration strategies can affect the deposition in the nasal epithelium and the delivery pathways to the CNS [8]. Successful N-to-B drug delivery requires maximization of the amount deposited on the olfactory epithelium. Thus, a novel nasal drug delivery device is another critical strategy for improving diagnosis and treatment effects, by directly transporting drugs from the nose to the brain. In clinical studies, nasal delivery devices can include: nose droppers, sprays, needleless syringes, breath-powered bi-directional nasal devices, pressurized meter dose inhalers, and pressurized olfactory delivery [8,9,18]. Droppers, spray pumps, and pipettes are conventional liquid nasal delivery methods and are less likely to target the olfactory via these systems due to the location of the olfactory epithelium in the upper part of the nose and the nasal cavity turbinate restriction, as well as the effect of changing of the head position [10,18]. Thus, the drug is either absorbed systemically by blood vessels or cleared by mucociliary clearance [10]. Therefore, researchers developed devices to deliver the drug in different forms (liquid/powder) to overcome the disadvantages of conventional nasal delivery systems. This section discusses some of these delivery systems and examples of their use in research. 

Djupesland et al. [22] reviewed the characteristics of the nasal delivery devices in general and the aerosol generation to achieve the clinical target of nasal drug delivery to the nose cavity, whether the substance in question has local action, is intended for systemic absorption, N-to-B transport, or a combination [22]. However, the focus is generally on how the nasal delivery devices could achieve the maximum dose deposition in the upper part of the nose cavity, thus targeting the brain. While liquid formulations are the oldest, cheapest, and most straightforward formulations, powder formulations are more stable, preservatives may not be required, and they often stick to the nasal mucosa before being dissolved and cleared [18]. Liquid-based delivery systems include catheter-delivered drugs, which are the simplest method of drug delivery to the nasal cavity by blowing with the mouth; the solution enters the nostril by the other end of the catheter in the nose cavity [18]. 

The ViaNase^TM^ is an electronic atomizer device used to target a nasal spray to the respiratory epithelia of the nasal cavity and the olfactory region [8,10]. This device consists of a sealed nosepiece and a device where an active vortex of nebulized particles is created; in humans, ViaNase™ is utilized to deliver insulin intranasally [9]. SipNose is an actuated nasal device to provide small particle aerosols with a minimum chance of deposition in the lower airways [9]. SipNose is applicable for various formulations: liquids and dry powders of small and large molecules, including biologics and cells [37]. Another device to enhance N-to-B drug delivery is Impel Neuropharma’s Precision Olfactory Delivery (POD^®^), which delivers either powder or liquids as aerosols. Instead of using the patient’s exhalation force, this device uses pressurized gas to emit the dose [6,9,10]. It contains a tank of compressed air or nitrogen, chlorofluorocarbon (CFC), or hydrofluoroalkane (HFA), used as a propellant and air chamber [18]; the manufacturer’s data claim a 50% deposition in the olfactory region [10]. 

As mentioned in the formulation section, powder an be administrated in a large dose and the solid-state prevents microbial contamination, hence the powder is preservative-free. The deposition of the powder and absorption of the drug from a powder formulation for nasal delivery depends on many factors, including the shape and size of powder particles, solubility, and flow characteristics [18]. The powder devices include Insufflators, Direct Haler, and Bi-Directional Optinose [18]. The Opt-Powder device by Optinose^®^ is a breath-actuated bi-directional delivery device that targets liquid or nasal powder formulations to the nasal cavity, including the olfactory region. In this device, the closure of the soft palate ensures that none of the flowing powder can be deposited into the lungs [8,9,22]; furthermore, it reduces the deposition in the lower nasal regions [10].

Other powder delivery devices are dry powder inhalers (DPI). Rhinocort Turbohaler^®^ (Budesonide), Teijin Puvlizer Rhinocort^®^ (beclomethasone dipropionate, BDP), Rhinicort Puvlizer^®^ (Budesonide), and Erizas^®^ (Dexamethasone Cipecilate) are popular nasal dry inhalers [18]. Aptar Pharma’s Unidose (UDS) Nasal Powder system is a DPI designed to deliver a single dose quickly, precisely, and easily [38]. Unidose-DPTM is a nasal powder sprayer developed by Bespak and resampled Flit Lizer technology; it contains a sealed container for delivering a single shot of a drug [18]. Delivery of antibody (human IgG) as a dry powder has been tested based on human MRI images in a nasal cast model. Bespak’s nasal cast model is a life-size model of the nasal cavity built from MRI images; it was used to determine the deposition pattern of drugs within the nose when delivered from Unidose-DP^TM^. As a result, 95% of the dose was delivered to the nasal cavity; most of it was deposited in the nasal vestibule, with only about 30% deposited into deeper regions of the nasal cavity [18,22]. SoluVent^TM^ is a powder delivery device developed by Beckton Dickinson (BD) [39]; vaccines have been given through this device which forces the powder to the nasal cavity [18]. Alchemy Pharmatech’s Naltos device works through an inert gas actuated by the device to propel the powder through the nares [6].

In general, efficient IN delivery to the CNS in humans can be achieved by optimizing the right combination of formulation delivery device, delivery volume, and head position to target specific nasal cavity regions [8]. In terms of selecting the formulation delivery device, in a human study with a whole-virus influenza liquid vaccine without adjuvant, the immune response was better when using the breath-powered Bi-Directional™ OptiNose device and nasal drops in the delivery, rather than traditional nasal spray and an oral spray [22]. In another study, Dong et al. [40] tested nasal cavities and aerosol delivery systems using an anatomical nasal model of a 60-year-old healthy male reconstructed from computed tomography images. The authors compared in detail the aerosol mask and breath-powered bi-directional systems using a computational fluid and particle dynamics approach. The results showed that the breath-powered drug delivery approach could produce superior olfactory deposition than the conventional aerosol mask approach [40]. In another study, the Optinose device was used to study the relationship between the effect of oxytocin (OT) peptide on social cognition with dose. The results showed a lower dose was needed with a breath-powered Optinose device to deliver OT across the nose to the brain. The lower doses were more efficacious in producing a cognitive response [18]; more examples of OT delivery are in the coming sections. The above examples show the importance of choosing a suitable nasal delivery device to deliver the drug efficiently to the CNS.

From the advanced nasal devices, only the Precision Olfactory Delivery, the Optinose, and ViaNase devices (which are advanced nebulizer devices) have been used in human N-to-B studies to date [6]. However, there is increased research on nasal drug devices for transporting and depositing the drug to the nasal cavity noninvasively, considering patient compliance. Thus, nasal delivery devices like powder devices, dry powder inhalers, bidirectional breath-powder delivery devices, meter-dosed spray pumps, nasal sprays, pressurized meter-dosed spray inhalers, nebulizers, atomizers, and pressurized olfactory devices have proceeded to clinical trials. The FDA has already approved some of the nasal drug delivery devices (mostly nasal spray) such as Zomig^®^, Astelin^®^, Narcan^®^, Dymista^®^, Advancia^®^, Onzetra™, and Nasonex^®^ to treat different disorders. Several patents on nasal delivery devices have been filed for delivering drugs to the brain; Pandey et al. [41] listed some patents. Furthermore, many biomedical device companies have obtained patents on novel delivery devices using technology like smartphones or a voice signal to trigger the release of the drug in a controlled manner [41]. Table 1 summarizes some nasal delivery devices with a brief description of the principles, dosage form, and limitations.

## 7. Nasal Liquid Formulated Compounds (Small Molecules, Peptides, and Proteins) for Therapy/Diseases Applications

The market value of nasal spray and inhalation generic drugs was $7.8 billion in 2018 and is predicted to reach close to $12 billion by the end of 2025 [42]. Most approved nasal formulations are liquid-based sprays that contain small-molecule drugs like sumatriptan to treat migraines and large molecules, including vitamin B12 and vasopressin [42]. Most nasal drugs that target CNS disorders are small molecules. Table 2 summarizes some of the current FDA-approved CNS nasal products. On the other hand, very few intranasally delivered large molecules are commercialized to treat CNS disorders. Agrawal et al. [43] listed some commercialized IN large molecules products, N-to-B transport of various proteins and peptides, and the various polymer and lipid-based nanocarrier systems used for N-to-B delivery of protein and peptides. Table 3 below lists some intranasally administered high molecular weight drugs, including peptides and proteins, for treating several disorders in the market.

Nasal sprays contain active pharmaceutical ingredients, for either systemic or local effects, in the form of a solution or suspension with excipients (e.g., preservatives, buffering agents, viscosity modifiers, emulsifiers), filled in non-pressurized dispensers that deliver a spray containing a metered dose of the drug. The dose is either metered by the spray pump or pre-metered during manufacture. Also, the nasal spray can be designed for unit dosing or discharge up to several hundred metered sprays of the formulation [24]. As mentioned in the IN-formulation section, some aspects of nasal sprays are unique and should be considered carefully during the development process (e.g., formulation, container closure system, stability, manufacturing, intermediates, and drug product). Any changes in the above aspects can affect the reproducibility of delivering the doses. Moreover, some of the unique features of nasal sprays are: metering and spray producing (e.g., orifice, nozzle, jet); energy is required for dispersion of the formulation as a spray by pushing the formulation through the nasal actuator and its orifice; and the design of the container closure system affects the dosing performance of the drug product [24].

Nebulizers use compressed gases (air, nitrogen, or oxygen) or ultrasonic or mechanical power to break the liquid formulation (solution or suspension) into a very fine mist that can be directly inhaled into the nose. As mentioned in the nasal device section, drugs delivered with conventional nasal nebulizer devices deposit in the nasal vestibule and nasal valve regions and do not reach the olfactory region [9,44]. Thus, new nebulizer devices have been developed to deliver drugs intranasally to the olfactory region. The advanced nebulizer devices include ViaNase™ (Kurve Technology, Inc., Lynwood, WA, USA), and precision olfactory delivery^®^ (POD^®^) device (Impel Neuropharma, Seattle, WA, USA).

**Table 2:** The current FDA-approved small molecule drug (non-protein/non-peptide) nasal products delivered to the CNS.

Nasal Product	Active Ingredients	Inactive Ingredients/Excipients	Dosage Forms and Strengths	Company	Indication	Approval Year	Reference(s)
Nicotrol	Nicotine	Sodium dihydrogen phosphate, disodium phosphate, methylparaben, propylparaben, edetate disodium, sodium chloride, polysorbate 80, citric acid, aroma, and water	The spray bottle contains 10 mg/mL of nicotine. Each actuation delivers a metered 50 µL spray containing approximately 0.5 mg of nicotine. One dose is 1 mg of nicotine (2 sprays, one in each nostril)	Pfizer INC	Smoking Cessation	1996	[45,46]
Migranal	Dihydroergotamine Mesylate	Caffeine anhydrous, dextrose anhydrous, carbon dioxide, purified water	Spray, Metered, Nasal. 0.5 mg/Spray	Bausch, Valeant Pharmaceuticals	Acute treatment of migraine headaches with or without aura	1997	[46,47,48]
Imitrex	Sumatriptan	Anhydrous dibasic sodium phosphate USP, monobasic potassium phosphate NF, sodium hydroxide NF, sulfuric acid NF, and purified water USP	Aqueous-buffered solution nasal spray Dosage: 5 mg, 10 mg, or 20 mg	Glaxosmithkline	Treatment of the symptoms of migraine headache and cluster headache	1997	[46,47,48,49]
Zomig	Zolmitriptan	Disodium phosphate dodecahydrate USP, citric acid anhydrous USP, and purified water USP	2.5 mg or 5 mg of zolmitriptan solution nasal spray in a 100 µL unit dose of aqueous buffered solution	Astrazeneca	Acute treatment of migraine in adults	2003	[46,47,48,49]
Narcan	Naloxone hydrochloride	Disodium ethylenediaminetetraacetate (stabilizer), sodium chloride, benzalkonium chloride (preservative), hydrochloric acid to adjust pH, and purified water	Solution metered nasal spray delivered as a single dose containing 2 mg or 4 mg of naloxone hydrochloride in 0.1 mL aqueous solution	Emergent, ADAPT Pharma, Inc.	Treatment of known or suspected opioid overdose	2015	[41,46,48,50,51]
Onzetra xsail	Sumatriptan Succinate	NA	Powder, EQ 11 mg Base. The recommended dosage is 22 mg (2 nosepieces)	Currax. Avanir Pharmaceuticals, Inc.	Acute treatment of migraine with or without aura in adults	2016	[46,47]
Tosymra	Sumatriptan	Potassium phosphate monobasic, citric acid monohydrate, n-Dodecyl beta-D-maltoside, sodium chloride, and sodium phosphate dibasic anhydrous in water	Each 100 uL of Tosymra solution nasal spray contains 10 mg of sumatriptan in a single-dose aqueous buffered solution.	Upsher Smith LabsDr. Reddy’s Laboratories Ltd.	Treatment of symptoms of migraine headache and cluster headache	2019	[41,46,52]
Spravato	Esketamine Hydrochloride	Sodium hydroxide, citric acid monohydrate, edetate disodium, and water for injection	The nasal spray device delivers two sprays containing a total of 28 mg of esketamine. Recommended Dosage: 56 mg or 84 mg	Janssen Pharms	Treatment-resistant depression (TRD) in adults	2019	[41,46,53]
Nayzilam	Midazolam	PEG-6 methyl ether, ethanol, polyethylene glycol 400, propylene glycol, and purified water	A single-dose nasal spray unit containing 5 mg of midazolam in 0.1 mL solution	UCB INC	Treatment of seizure activity	2019	[41,46,54]
Valtoco	Diazepam	Dehydrated alcohol, benzyl alcohol (10.5 mg per 0.1 mL), n-dodecyl beta-D-maltoside, and vitamin E	Nasal spray solution that available in 5 mg, 7.5 mg, and 10 mg strengths and contains 0.1 mL solution	Neurelis INC	Short-term treatment of seizure clusters	2020	[41,46,47,55]
Numbrino	Cocaine Hydrochloride	Sodium benzoate, citric acid (anhydrous), purified water, D&C Yellow No. 10, and FD&C Green No. 3	Available in 4% strength (400 mg/10 mL). Each 1 mL contains cocaine hydrochloride 40 mg (35.7 mg of cocaine-free base)	Cody Labs INC	Induction of local anesthesia	2020	[41,46,56]
Stadol	Butorphanol tartrate Butorphanol tartrate	Sodium chloride, citric acid (anhydrous), benzethonium chloride, purified water, and sodium hydroxide	Nasal spray solution, Metered. 1 mg/spray	Bristol Myers Squibb	Pain management Nicotrol	1991	[46,57]
Butorphanol tartrate		Sodium citrate, dihydrate, citric acid hydrous, andsodium chloride		Apotex Corp., Mylan Pharmaceutical Inc., Hikma Pharms		2001, 2002	[46,48,58]
Goprelto	Cocaine hydrochloride	Sodium benzoate, anhydrous citric acid, D&C Yellow No. 10, FD&C Green No. 3, and purified water.	Nasal solution, 4% strength	Genus Lifesciences Inc.	Induction of local anesthesia of the mucous membranes	2017	[46,48,59]

**Table 3:** A list of intranasally administered high molecular weight drugs including peptides and proteins on the market.

Peptide Name	Product Name (Manufacturer)	Strength	Inactive Ingredients	Dosage Form	Disease Application	Approval Year	Reference(s)
Desmopressin acetate (Peptide drug)	DDAVP (Ferring Pharmaceuticals Inc., Parsippany, NJ, USA)	0.01 mg/spray	Disodium phosphate dihydrate, citric acid monohydrate, sodium chloride, and benzalkonium chloride solution (50%)	Solution, spray, metered	Antidiuretic and Vasopressor Hormones	1978	[46,48]
	Concentraid (Ferring Pharmaceuticals Inc., Parsippany, NJ, USA)	0.01 mg/spray	NA	Solution, spray	To determine the extent of renal impairment in children with urinary tract disorders	1990	[46]
	Octostim Spray (Ferring Pharmaceuticals Inc., Parsippany, NJ, USA)	1.5 mg/mL	Disodium phosphate dihydrate, citric acid monohydrate, benzalkonium chloride (preservative), purified water, and sodium chloride	Spray	Prevent bleeding for people with mild hemophilia A and mild von Willebrand’s disease type I. Treat central diabetes insipidus.	1998	[43,60]
	Stimate (Ferring Pharmaceuticals Inc., Parsippany, NJ, USA)	0.15 mg/spray	Sodium chloride, citric acid monohydrate, disodium phosphate dihydrate, benzalkonium chloride, and purified water	Solution, spray, metered	Hemophilia A, von Willebrand’s Disease (Type I)	1994	[46,48]
	Minirin^®^ (Ferring Pharmaceuticals Inc., Parsippany, NJ, USA)	0.01 mg/spray	Chlorobutanol, sodium chloride, and hydrochloric acid to adjust pH to approximately 4	Solution, spray, metered	Primary nocturnal enuresis, central cranial diabetes insipidus	2002	[43,46,48,61]
	Desmopressin acetate USP spray solution (Apotex Inc., Toronto, ON, Canada)	0.01 mg/spray	Benzalkonium chloride solution (50% *w*/*v*), citric acid monohydrate, Sodium chloride, Sodium phosphate dibasic heptahydrate, and purified water	Solution, spray	Primary nocturnal enuresis, central cranial diabetes insipidus	2005	
	Noctiva (Roivant, Avadel Pharmaceuticals, Serenity Pharmaceuticals, Milford, PA, USA)	0.00083 mg/spray or 0.00166 mg/spray	Citric acid monohydrate, sodium citrate dihydrate, cyclopentadecanolide, cottonseed oil, sorbitan monolaurate, polysorbate 20, and water for injection	Oil-in-water emulsion, spray, metered	Nocturia	2017	[46,48]
	Desmospray	0.01 mg/spray	Disodium phosphate dihydrate, citric acid monohydrate, sodium chloride, benzalkonium chloride solution 50%, and purified water	Solution, spray	Cranial diabetes insipidus or nocturia associated with multiple sclerosis		[61,62]
	OCTIM	0.15 mg/spray	Disodium phosphate dihydrate, citric acid monohydrate, sodium chloride, benzalkonium chloride solution 50%, and purified water	Solution, spray	Octim Nasal Spray used: In patients with von Willebrand’s disease or hemophilia. In testing fibrinolytic response		[63]
Buserelin acetate (Protein-based therapies-hormones)	Suprefact™ (Hoechst Canada Inc., Montreal, QC, Canada)	IN solution 1 mg/mL	Benzalkonium chloride, citric acid/sodium citrate buffer, and sodium chloride	Solution, spray,	Prostate cancer, endometriosis	1988	[43,61,64,65]
	Suprecur^®^ (Sanofi Aventis, Paris, France)	0.15 mg/spray	Citric acid, sodium citrate, sodium chloride, and benzalkonium chloride in aqueous solution	Solution, spray	Prostate cancer, endometriosis	2002	[61]
Nafarelin acetate	Synarel^®^ (Pfizer, New York, NY, USA)	0.2 mg/spray	Glacial acetic acid, sodium hydroxide or hydrochloric acid (to adjust pH), benzalkonium chloride, sorbitol, and purified water	Solution, spray, Metered	Endometriosis, central precocious puberty (CPP)	1990	[46,48,61]
Gonadorelin, LHRH (Gonadotropin-releasing hormone agonist)	Kryptocur^®^	0.2 mg/spray	NA	Solution, spray	Cryptorchism	1982	[61,64]
Salmon calcitonin (Protein-based therapies- hormones)	Miacalcin^®^ (Mylan Inc., Canonsburg, PA, USA)	200 IU/spray	Benzalkonium chloride, sodium chloride, hydrochloric acid (added as necessary to adjust pH), and purified water	Solution, spray, Metered	Postmenopausal osteoporosis	1995	[43,46,49,61,65]
	Fortical^®^ (Unigene Laboratories, Inc., Boonton, NJ, USA), (Upsher Smith Labs)	200 IU/spray	Citric acid, phenyl ethyl alcohol, sodium chloride, benzyl alcohol, polysorbate 80, hydrochloric acid or sodium hydroxide (to adjust pH), and purified water	Solution, spray, Metered	Postmenopausal osteoporosis	2005	[43,46,48,61,66]
OT (Peptide)	Syntocinin™ (Novartis, Basel, Switzerland)	40 IU/mL	NA	Solution, spray	Indicated for the initiation or improvement of uterine contractions	1960	[61,64]
Protirelin (synthetic analogue of thyrotropin-releasing hormone (TRH))	Antepan^®^ (Sanofi Aventis, Paris, France)	1 mg/0.09 mL	NA	Solution, spray	Hypothyroidism and acromegaly	1984	[61,64,67]
Four vaccine virus strains: an A/H1N1 strain, an A/H3N2 strain and two B strains	FluMist^®^ Quadrivalent (MedImmune, LLC., Gaithersburg, MD, USA)	10^6.5–7.5^ FFU (fluorescent focus units) of live attenuated influenza virus reassortants of each of the four strains in 0.2 mL dose	Monosodium glutamate, hydrolyzed porcine gelatin, arginine, sucrose, dibasic potassium phosphate, and monobasic potassium phosphate	Suspension, spray	Active immunization for the prevention of influenza	2013	[49,61,64]
Glucagon (Protein based therapies-hormones)	Baqsimi (Eli Lilly & Co., Ltd., Basingstoke, UK)	3 mg	Betadex, and dodecylphosphocholine	Powder	Severe hypoglycemia	2019	[43]
Cyanocobalamine (man-made form of vitamin B12)	Nascobal^®^ (Par Pharm Co., Irvine, CA, USA)	0.5 mg/INH	Citric acid, glycerin, sodium citrate, and benzalkonium chloride in purified water	Gel, Metered; Nasal	Treat low blood levels of vitamin B12	1996	[46,65,68]
	Nascobal^®^ (Endo Pharms Inc., Dublin, Ireland)	0.5 mg/SPRAY		Spray, Metered; Nasal		2005	[46,65,69]

Insulin has emerged as a key regulatory hormone in the central nervous system (CNS). Insulin receptors in the brain are located mainly in synapses and can be found in the hippocampus, entorhinal cortex, and frontal cortex, where insulin signaling leads to synaptogenesis and synaptic remodeling [70]. Changes in brain insulin metabolism and insulin resistance are associated with several CNS disorders such as Alzheimer’s disease (AD), depression [71,72,73], autism [74], schizophrenia [75], Huntington’s disease [76], Parkinson’s disease (PD) [77], etc. IN-insulin has shown an improvement in memory, metabolic integrity of the brain in patients with AD, and mild cognitive impairment [78]. A study by Craft et al. [79] examined the effects of IN insulin administration on cognitive and functional outcomes in adults with AD. In this study, insulin was administered intranasally at a dose of 20 IU or 40 IU for 4 months using a ViaNase nasal drug delivery device (Kurve Technology, Bothell, Washington). This device released a metered dose of insulin into a chamber covering the participant’s nose, which was then inhaled by breathing regularly for 2 min until the prescribed dose was delivered. IN delivery of insulin showed improved delayed memory compared to the placebo, and these results suggest that IN insulin may have beneficial effects in adults with AD. Several other studies also studied the effect of IN insulin delivered using the nebulizer device ViaNase and observed improvements in cognition, functionality, cortical blood flow, and vasoreactivity [80,81,82,83]. In a study by Craft et al. [84], POD^®^ device has shown good reliability in delivering a specified dose of insulin to the olfactory cleft compared to the ViaNase device. 

**Figure 2:**
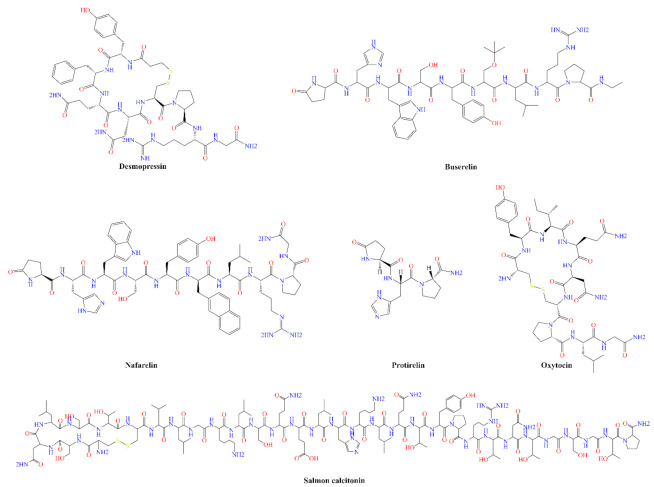
Chemical structures (drawn using CambridgeSoft™, Cambridge, MA, USA) of example peptides used in nasal delivery systems.

Oxytocin, a nine amino acid neuropeptide, plays an important role in social behavior [85,86,87,88]. It is produced by the paraventricular and supraoptic nuclei of the hypothalamus and released into the bloodstream via the portal circulation of the posterior pituitary [89]. It is one of the most studied peptides for N-to-B targeting in CNS disorders [90,91,92,93,94]. In human studies, IN administration of OT showed increased trust [95], positive communication [96], reduce cortisol and increase anxiolytic effects in stress [97], improved mind reading [98], and has a positive effect on social behaviors in both normal [99] and autistic persons [100,101]. Dal Monte et al. [102] evaluated the OT levels in CSF and blood after IN administration (48 IU OT) to macaques using either a spray or a nebulizer device. Both spray and nebulization of OT resulted in increased levels of OT in CSF. However, the blood OT levels were higher after nasal spray compared to the nebulization. Figure 2 shows the chemical structures of some peptides used in nasal delivery systems.

## 8. Nasal DPI Peptides for CNS Therapy/Disease Application

The IN delivery of proteins and peptides to the CNS allows for the treatment of neurodegenerative diseases such as AD [103], PD [104,105,106], and multiple sclerosis [107,108], among others. However, despite many published articles using the N-to-B route to target peptides and protein to the CNS, nearly all use liquid formulations like emulsions, suspensions, and sprays, among others, and only a few use formulations in solid-state.

Due to their higher medication stability and lack of preservatives and propellants, nasal dry powders are desirable formulations [18,22]. Fransén, et al. [109] proved that IN delivery of desmopressin peptide to the brain tissue was more efficient as a nasal powder, showing superior bioavailability than commercial nasal liquid spray and sublingual tablet. Despite their advantages, nasal powders have yet to gain acceptance on the market, and only a few published articles on nanoparticles (NPs) targeting the CNS can be found. This section discusses the nasal powder administration of peptides and proteins intended to be N-to-B to treat CNS disorders, giving more focus in recent years.

Salade, et al. [110] used N-to-B delivery of ghrelin loaded in lipid nanoparticles as a suitable strategy for cachexia management. Ghrelin (GHRL) is a 28-amino acid cationic peptide hormone that binds to growth hormone secretagogue receptors, releasing neuropeptide Y and Agouti-related peptide (AgRP), subsequently stimulating food intake and decreasing inflammatory cytokine levels. Developing a dry powder of Chitosan-coated liposomes loaded with ghrelin, as opposed to a liquid formulation, is appealing due to the drug’s better stability during long-term storage, increased mucoadhesion, and enhanced permeability through biological membranes. The powder formulation was loaded into a unit-dose system device (UDS) (Aptar Pharm, LeVaudreuil, France); the nasal device guarantees the olfactory region, which is the primary target for N-to-B transfer, is targeted. Moreover, more than 50% *w*/*w* was deposited in the olfactory zone using a 3D printed nasal cast. Deposition in this region was maximized using nasal powders instead of liquid GHRL formulation [110].

In another study, Zada, et al. [111] targeted N-to-B delivery of thyrotropin-releasing hormone (TRH)(a polypeptide used for treating suicidal patients), using fast degrading poly(sebacic anhydride) (PSA) NPs. The NPs can be delivered to the olfactory epithelium using a refillable nasal atomizer that deposits NP mist to the olfactory neuroepithelium [111]. Godfrey, et al. [112] showed that the use of IN NPs delivery system enables the administration of a selective opioid receptor (DOR) agonist, leucine-kephalin hydrochloride (LENK), to the brain. Animals that were dosed with NPs developed mild to reduced pain. According to the researchers, these NPs might be offered as a microparticle-based powder to meet regulatory standards. At the dose administered, the nanoparticle polymer was well tolerated via the nasal route. [112].

As reported by von Mentzer, et al. [113], they produced a nasal powder of poorly bioavailable synthetic N-terminally truncated and modified human calcitonin gene-related peptide (CGRP) (^34^Pro,^35^Phe)CGRP_27–37_ by spray drying a solution with 1% chitosan as a mucoadhesive polymer for enhancing N-to-B delivery to treat migraines. The researchers reported that administering the peptide to mice reduced CGRP-induced plasma protein extravasation (PPE), indicating the antagonistic effects of the peptide in vivo. Thus, this peptide, which is a potent CGRP receptor antagonist, can be formulated as a dry powder with no loss of activity, indicating its potential as a nasally formulated anti-migraine medicine. Nasally delivered tiny peptides offer a viable, cost-effective, and patient-approved alternative to continuous CGRP blocking without cardiovascular hazards [113].

## 9. Clinical Trials of IN Administration of Peptides for CNS Therapy

Although N-to-B delivery has been well established in clinical trials, improved N-to-B delivery systems have yet to be associated with approved products [6]. The clinical development of peptide N-to-B formulations is still in its early stages, which is understandable given the small number of animal trials. Nonetheless, some peptides/proteins have advanced through drug development, as shown in Table 4. Some of them, such as insulin and OT, have completed Phase IV clinical trials more recently. Insulin, OT, and hypocretin-1 are the most studied products to be delivered to the brain through this direct path. Because of the broad distribution of their receptors in the CNS, these well-characterized peptides have critical controlling roles. In addition, several clinical trials have shown their potential in treating various disorders, including cognitive and behavioral disorders, narcolepsy, and other significant neurological disorders, such as AD and PD, that have been reviewed extensively elsewhere [16]. Therefore, we will highlight some of the clinical trials, focusing more on the recent ones in this review.

In a recent clinical trial, Craft et al. [84] studied the feasibility and efficacy of IN insulin in persons with mild cognitive impairment and AD, finding no improvements in cognitive or functional benefits compared with placebo after 12 months of daily dose. They suggest a need for better delivery devices to ensure the increase of insulin levels in the CNS, which might determine the therapeutic effect in persons with CNS disorders [84].

Even when virologically suppressed, almost half of HIV-positive people on antiretroviral therapy have moderate neurocognitive dysfunction (HIV-NCI). In this sense, Rosenbloom, et al. [114] conducted a single-center, randomized, double-blind, placebo-controlled study to compare the efficacy of IN glulisine (fast-acting insulin) vs. placebo in N = 35 participants with memory impairment and Alzheimer’s disease. They observed that insulin was well tolerated but did not positively affect cognition, function, or mood after IN administration. They conclude that a larger study population is necessary to evaluate rapid-acting insulin efficacy in this population better. The same research group has registered a clinical trial for IN Insulin in Frontotemporal Dementia (FTD) in 12 patients, which is critical, as no treatment medicines for the advancing cognitive symptoms of FTD have been developed. Novak, et al. [115], aimed to determine the effects of IN-insulin on cognition and motor performance in PD. Preliminary results show that after four weeks of regular IN therapy, functional skills improved, paving the way for a larger cohort study to assess the long-term safety and possible effectiveness of IN-insulin administration to treat and prevent functional decline in PD patients.

Reger et al. [116] conducted research to see if delivering insulin into the brain of people with Alzheimer’s disease or amnestic moderate cognitive impairment (MCI) would improve memory without increasing plasma insulin levels. With a needle-free syringe, 100 µL of insulin or saline were injected into alternate nostrils for a total administration volume of 400 µL. After receiving the insulin, the subjects were asked to sniff to aid in the delivery of the drug into the nasal cavity. The study findings suggested that IN-insulin administration may benefit significantly without the risk of peripheral hypoglycemia [116]. Another study of the same group, Reger et al. [80] investigated at the cognitive dose-response curves related to IN-insulin delivery in persons with AD or amnestic MCI. They examined the effect of IN-insulin treatment on plasma Aβ, since peripheral insulin delivery modifies plasma amyloid (Aβ) levels. They expected that IN-insulin treatment will improve memory and regulate Aβ, with dose-response curves differing depending on apolipoprotein (APOE) genotype. [80]. They used the same insulin or saline as in their previous study [116], and their findings revealed that individuals with varying genetic risks for AD may have variable dose-response curves after receiving IN-insulin [80].

Hollander, et al. [117] studied the use of IN OT in children with Prader–Willi Syndrome (PWS). They found that placebo was associated with modest improvement in hyperphagia and repetitive behaviors in childhood PWS, whereas IN OT was not associated with improvement in these domains. Russell, et al. [118], studied the use of IN OT as a treatment for Anorexia Nervosa (AN), an illness marked by high anxiety, social behavior deficit, and withdrawal, because evidence suggests that OT can improve this condition [119,120,121]. Salivary cortisol levels were considerably lower in anticipation of an afternoon snack after four weeks of IN OT compared to placebo. In self-administered 36 IU IN OT patients, morning plasma OT levels did not change following chronic IN OT or with weight restoration. In another study, Ma, et al. [122] examined the role of OT in belief updating upon desirable and undesirable feedback. The IN OT-induced deficiency in belief updating in response to unfavorable feedback was more pronounced in people who have a high level of depression or anxiety than in people who have a low level.

Guastella et al. [90] looked at the impact of the OT peptide on emotion detection in children with autism spectrum disorders. They reasoned that nasal OT spray could provide a quick, safe, and effective treatment for emotion recognition impairments. They hypothesized that nasal OT spray might lead to a brief, safe, and effective intervention to remedy emotion recognition deficits. Participants received a single dose of OT and a placebo nasal spray one week apart [90]. The study’s results demonstrated the first evidence that nasal OT spray improves emotion recognition in young people diagnosed with autism spectrum disorders. Therefore, earlier intervention and more investigation of nasal OT spray as medication may be possible to improve social communication in youth with autism spectrum disorders [90].

Arginine vasopressin (AV) is a neurotransmitter that is important for social interaction and observational learning. Parker, et al. [123] probed the effectiveness of vasopressin nasal spray for treating symptoms associated with autism in children 6–12 years old. Intranasal AV treatment, compared to placebo, enhanced social abilities, diminished anxiety symptoms, and decreased repetitive behaviors. AV has also been studied for enhancing cooperative behavior in Schizophrenia. Findings revealed that single-dose administration of 40 IU of AV did not significantly improve risky cooperative behavior in schizophrenia patients. However, they conclude a long-term study with repeated administration is needed for a definitive conclusion [124]. In an ongoing clinical trial at the University of Electronic Science and Technology of China, they are investigating whether IN AV (20IU) could influence attention control in a social-emotional saccade/antisaccade eye-tracking paradigm. Early results show that IN AV enhances attention to social signals by lowering the impacts of competing goal-directed top-down attention and enhancing the importance of bottom-up processing, as evidenced in the current study utilizing an emotional anti-saccade task [125].

Baier et al. reported a study on Narcolepsy patients to see if the olfactory function is decreased in the well-defined narcolepsy group with cataplexies, compared to healthy matched controls. Another goal of the study is to discover if this malfunction is related to an orexinergic deficiency and whether orexin-A may be used to restore it. Orexin-A is a neuropeptide that was abnormally decreased or undetectable in the CSF of a large proportion of patients with narcolepsy. Subjects were given the nasal spray Orexin- A (or placebo) via a hand-operated pump that sprayed a thin mist into their nostrils. For 10 min, 0.1 mL of fluid was delivered into each nostril at 1 min intervals, resulting in a total volume of 2 mL. This study found that IN orexin-A administration essentially restores olfactory performance. Hence, there is evidence for the hypothesis that pathophysiological mechanisms underlying olfactory dysfunction are directly caused by a lack of orexin-A [126].

Zhao et al. [127] synthesized phospholipid-based gelatin nanoparticles encapsulating basic fibroblast growth factor (bFGF) for nasal delivery to the brain. Nanoparticles containing bFGF (5 µL) were pipette-administered to alternate nostrils of deeply sedated rats in this investigation. The researchers successfully increased bFGF brain supply via the non-invasive IN route, and hemiparkinsonian rats demonstrated a considerable neuroprotective improvement [127].

The systemically acting intranasally administered biologics (proteins and peptides) under clinical investigation as enrolled at clinicaltrials.gov was listed by Rohrer et al. [64]. Furthermore, the current status of clinical trials (collected from clinicaltrials.gov) of the N-to-B delivery systems for several CNS disorders/diseases, including proteins and peptides, was discussed by Pandey et al. [41]. Several patents are available for drug delivery via the nasal route to either directly target the CNS, or by systemic transport of the drugs by the nasal route to target the CNS. These patents include IN drug delivery systems, formulation composition, devices employed, and the care of numerous CNS-related disorders, among other things. Patents for IN delivery of new formulations to the CNS, patents for IN delivery of peptides/proteins to the CNS, patents explaining delivery devices for IN delivery to the CNS, and patents for IN delivery of synthetic drugs to the CNS are some of the types of patents filed on IN delivery to the brain. [41].

**Table 4:** Peptide/Proteins drugs in clinical trials for N-to-B delivery.

Peptide/Protein	Clinical Trial	Disease/Condition	Device/Dose	Reference(s)
Insulin	NCT01767909	Insulin (Humulin^®^ R U-100) for mild cognitive impairment and AD	Metered dose inhaler: ViaNase; Kurve Technology; 20 IU bid taken twice daily	[41,84,114,115,128]
	NCT02503501	Mild cognitive impairment and AD	Impel NeuroPharma I109 Precision Olfactory Delivery (POD^®^) device	
	NCT01595646	Insulin detemir (Levemir) to evaluate its effect on diseased Alzheimer patients	IN	
	NCT02462161	Insulin aspart: to evaluate the safety Alzheimer’s Disease, Mild Cognitive Impairment	IN	
	NCT01547169	Insulin detemir (Levemir): to evaluate its effect on diseased Alzheimer patients	IN	
	NCT04115384	Frontotemporal Dementia	Non-stated; Regular insulin (Novolin-R) 20 IU/IN (0.1 mL/10 units IN in each nostril)	
	NCT02064166	Parkinson’s	Via Nase device Kurve Technology; 40 IU of IN insulin daily	
OT	NCT03197662	Prader–Willi syndrome	Non-stated; 16 IU of IN-OT (8 IU per nostril)	[41,117,118,122]
	NCT03414112	Anorexia Nervosa	Amber 7 mL glass nasal spray with a metered pump (Bondi, NSW); each pump spray delivered 50 μL of OT (9 IU)	
	NCT03597893	social adaptation―in belief	Nasal spray (Syntocinon^®^); single IN dose of 24 IU OT	
	NCT02508103	To study the effect of drug on Premenstrual dysphoric Disorder	Nasal spray	
	NCT02567032	OT (Syntocinon) for Schizophrenia: to evaluate the safety and efficacy	Nasal spray	
	NCT03245437	OT with social cognitive skills Training: to evaluate the efficacy	Nasal spray	
Arginine Vasopressin	NCT03204786	Autism	Amber glass bottles with metered (0.1 mL per puff) nasal spray applicators; 4 IU twice daily	[123,124,125]
	NCT04190004	Schizophrenia	Non-stated; IN 40 IU once/day	
	NCT04493554	Attention Control	Non-stated; IN 20 IU	

## 10. Conclusions

Neuropeptides are vital in the function of the CNS, and their receptors have high potential as targets to treat several CNS diseases. The development of peptides as therapeutics has been limited by the lack of drug-like properties of peptides. Recent technical advances have overcome many peptide delivery limitations and led to rapid growth in developing therapeutic peptides for many diseases.

Researchers made many efforts to develop the delivery of the drugs in general and in peptides specifically across the BBB, including modifying therapeutic agents, altering the barrier, carrier-mediated transport integrity, invasive techniques, etc. However, opening the barrier by such means allows entry of xenobiotics circulating in the blood to the CNS. That forced an alternative approach focused on bypassing the BBB by using the non-invasive N-to-B delivery. The nasal mucosa-brain neural connections give a unique pathway for delivering therapeutic agents, including peptides, to the CNS, eliminating systemic delivery, and reducing systemic side effects. However, the mechanism underlying transferring the drugs to the brain has not been completely elucidated. Generally, N-to-B delivery does not require modification of therapeutic agents or coupling with carriers. However, as a new approach to enhancing the stability of peptides specifically, and as a part of developing new technologies for the protection and delivery of peptides, the glycosylation strategy, by attaching a sugar moiety to the peptides, is now gaining more attention. The preliminary results from research laboratories (including ours) showed an enhancement in the pharmacokinetic and pharmacodynamic parameters of the glycopeptides compared to their counterpart peptides.

Another aspect to consider is the formulation, to design and tailor appropriate IN drug formulations. The physicochemical characteristics, the cytoarchitecture, and the mucociliary clearance of the nasal mucosa are essential criteria. Besides the commonly used liquid formulation, the development and engineering of dry powders using a spray dryer look attractive due to their higher long-term storage stability, increased mucoadhesion, and enhanced permeation across biological membranes. Furthermore, the powder obtained low residual moisture and engineered particle and particle surface morphology, particle size, and particle size distribution, tailored for nasal delivery.

The nasal delivery device is an essential factor to consider during the early stage of the development process; it is rare that any medical devices on the market that can specifically target only one delivery pathway. Many studies in human and animal models suggested that the deposition of the drugs sprayed by some nasal devices was higher in the olfactory region than the deposition of the drugs sprayed by traditional Nasal sprays. In addition, higher drug concentration was also noticeable in the brain areas. Again, these studies indicate that selecting the suitable device can effectively deliver therapeutics to the brain, bypassing the BBB. Future prospects of developing nasal devices will lead to developing new types of CNS peptide drugs, expanding the number of potential clinical compounds, and improving existing drugs’ safety.

Nasal delivery is an effective delivery strategy. There continues to be a significant increase in the number of commercially available pharmaceutical nasal inhalation products (including protein and peptides) for clinical use by patients for CNS therapeutic applications. These include nasal inhalation delivery for non-invasive brain therapy for rescue therapy of acute migraine attacks, rescue therapy for heroin and opioid overdose, and neurodegenerative diseases such as Alzheimer’s. There is an increased interest and potential in this exciting area of research, and the future looks bright for nasal inhalation delivery for CNS and brain therapeutic applications, hand in hand with the development of peptides as drug candidates, to be delivered intranasally.

## Figures and Tables

**Table 1:** Examples of Nasal Delivery Devices with a brief description of the principles, dosage form, and limitations.

Delivery Devices	Description	Deposition Region	Dosage Form	Reference(s)
Nasal drops	Delivered with glass dropper or pipette Cleared faster than nasal sprays. Efficacy can be affected by patient administration technique. Microbial contamination and chemical instability	Spread over a larger area than nasal sprays. More deposition in the olfactory region compared to using nasal sprays	Liquid	[9]
Meter-dosed pump sprays	Deliver between 25 and 200 μL per spray. Relatively easy for patients to use Reproducible delivered doses Expensive Efficacy can be affected by patient administration technique Preservatives are required to prevent contamination.	Deposition from pump sprays is mostly to the anterior regions of the nasal cavity, encompassing the vestibule and nasal valve area 2.5% is deposited in areas corresponding to the olfactory region	Liquid	[9,22]
Catheter-delivered drugs	Simple method Require anesthesia to deliver the liquid Mucosa is sensitive to the site of deposition Not suitable for self-administration	Drug delivery to the nasal cavity by inserting a catheter in the nose cavity as drops or as a “liquid jet.”	Liquid	[18]
SipNose	Actuated nasal device Non-invasive High compliance	More localization of aerosolized drug in the trigeminal nerve and/or the olfactory epithelium. More delivery of small particle aerosols with less deposition in the lower airways.	liquid and powder	[9]
ViaNase^TM^ (Developed by (Via- Nase by Kurve Technology Inc., Lynnwood, WA, USA).	Vortex-propelled nebulizer device with a sealed nosepiece and an active vortex creates the nebulized particles. Generate droplets with a diameter of 9–11 µm [10] Lung deposition of insulin delivered with this device is likely to occur [22]	Delivers liquid droplets (15–20 μm in size) to the nasal cavity, including the olfactory region. Maximize deposition to the olfactory and transport to the brain	Liquid	[6,8,9,10,22]
Insufflator	Composed of straw or tube with drugs. Local anesthetics or decongestants are needed before insufflations delivery. Breath activated	Directly delivers drugs to the olfactory region	Powder	[18]
Optinose^®^ (Opt- Powder device) Bi-Directional^TM^ Breath Powered^®^ DDSs	Bi-directional delivery device Consists of: sealing nosepiece, mouthpiece, conical, pump-spray, and an additional breath-activated mechanism to create 43-µm droplets [10]	Directly delivers drugs to the olfactory region, thus, delivery into the brain, circumventing the blood-brain barrier Produce reliable dosing	Liquid and powder	[9,10,18]
Direct Haler (Designed by a Danish company)	Free from contamination and preservatives, priming, and cleaning. Delivering fine particles into the nasal cavity with lungs exposure	Delivers fine particles into the nasal cavity with lungs exposure	Powder	[18]
Precision Olfactory Device (POD) Impel Neuropharma’s	Deliver powder or liquids through an insufflation method Propellant activated (use pressurized gas to emit the dose instead of using the patient’s own exhalation) Patient’s ease of use	Delivers aqueous liquids and powders targeting the vascular rich upper nasal space, thus, the brain	Liquid and powder	[6,9,18]
Alchemy Pharmatech’s Naltos Device	Unit-dose, needle-free IN delivery propellant activated (works by means of an inert gas that is actuated by the device to propel the powder through the nares). Originally developed for drug powder formulations and can be used for peptides and vaccines.	Delivers medicines across the nasal mucosa into the bloodstream	Powder	[6]
Dry powder inhalers (DPI)	simple in design, cheap, can be operated without medical supervision	Doses range from µg to mg	Powder	[18]
Unidose-DP^TM^	Delivering a single shot of a drug. Air-filled compartment which kept compressed until a pin ruptures a membrane to release the pressure to emit the powder [22]	About 95% of the drug was delivered to the nasal cavity, and 60–70% of it reached the nasal vestibular region	Powder	[18]
SoluVent^TM^	A plunger creates a positive pressure by piercing a membrane to expel the powder [22]	Nasal cavity	Powder	[18]

## Data Availability

The data presented in this study are available on request from the Corresponding Author.
